# Yeast osmoregulation – glycerol still in pole position

**DOI:** 10.1093/femsyr/foac035

**Published:** 2022-08-04

**Authors:** Anders Blomberg

**Affiliations:** Department of Chemistry and Molecular Biology, University of Gothenburg, 405 30 Gothenburg, Sweden

**Keywords:** yeast, osmoregulation, osmo-sensing, osmo-signaling, cell cycle delay, gene expression

## Abstract

In response to osmotic dehydration cells sense, signal, alter gene expression, and metabolically counterbalance osmotic differences. The main compatible solute/osmolyte that accumulates in yeast cells is glycerol, which is produced from the glycolytic intermediate dihydroxyacetone phosphate. This review covers recent advancements in understanding mechanisms involved in sensing, signaling, cell-cycle delays, transcriptional responses as well as post-translational modifications on key proteins in osmoregulation. The protein kinase Hog1 is a key-player in many of these events, however, there is also a growing body of evidence for important Hog1-independent mechanisms playing vital roles. Several missing links in our understanding of osmoregulation will be discussed and future avenues for research proposed. The review highlights that this rather simple experimental system—salt/sorbitol and yeast—has developed into an enormously potent model system unravelling important fundamental aspects in biology.

## Teaser

Yeast osmoregulation is a multifaceted response that integrates sensing, signaling, cell cycle delay, transcriptional changes, and metabolic adjustments.

*‘Striving for an integrative view*’ (Hohmann, 2002)

## INTRODUCTION AND SOME PHYSIOLOGICAL BACKGROUND

Osmoregulation is the ability of cells to sense, signal and metabolically counterbalance osmotic challenges in their surrounding (Hohmann *et al*. 2007). Yeasts are exposed to highly variable environments in nature where the water activity can range widely and change rapidly (Blomberg and Adler 1992). Decreased external water activity causes water to follow the concentration gradient and diffuse out of cells, which results in that cell volume decreases. However, the yeast cell volume reaches a plateau at around 40%–50% of the initial volume for very high concentrations of external solutes (e.g. NaCl or sorbitol), thus apparently not being able to shrink further (Schaber *et al*. 2010, Miermont *et al*. 2013). The initial cell-shrinkage of yeast is a rapid process where dehydration is completed in less than a minute, a consequence of the high water-permeability of biological membranes and the presence of water channels, aquaporins (Hohmann *et al*.^2007^). Living cells maintain an osmotic gradient between their interior and the extracellular environment. This osmotic gradient is counterbalanced by a hydrostatic pressure, turgor, which is especially prominent in plants and unicellular organisms (Rojas and Huang 2018). A systematic study of the yeast biophysical properties reported that the yeast cell wall is quite elastic since it tightly follows the cell membrane on shrinkage i.e. plasmolysis only occurs at very high levels of stress (Schaber *et al*. 2010). Scanning electron microscopy studies on yeast have highlighted dramatic morphological changes during the early stages of dehydration (Saldana *et al*. 2021). Within a couple of seconds after exposure to high salt yeast cells shrink and display a wrinkled surface. However, these morphological changes are completely reversible upon return to pure water where cells rapidly again acquire a smooth and ellipsoid shape, regaining turgor, and showing that the yeast cell wall is remarkably elastic and flexible.

Osmotic dehydration also leads to an increase in intracellular protein concentration, and this molecular crowding has important consequences for cellular processes. Evolution has possibly selected for optimal protein density, which originates from a balance between two counteracting effects on biochemical kinetics: a positive effect at high protein concentrations from enhanced probability of protein-protein interactions and protein-ligand associations, and a negative effect from high protein concentrations because of slow-down of diffusion in an overcrowded cytoplasm (Dill, Ghosh and Schmit 2011). Indeed, a roughly 10-fold decrease in diffusion coefficient has been reported after osmotic dehydration, from ∼15 μm^2^s^−1^ in basal conditions to ∼1.7 μm^2^s^−1^ in 1 M sorbitol (Miermont *et al*. 2013). In addition, the kinetics of a number of cellular processes are reduced as a consequence of osmotic dehydration and crowding i.e. slow down of the nuclear translocation of diverse transcription factors, the mobility of an actin-binding protein, vesicular trafficking, and endocytosis.

The two most applied stress-agents in studies of osmoregulation in yeast are NaCl and the six-carbon polyol sorbitol. The osmotic effect *per se* is largely molecule-unspecific and almost exclusively relates to the ‘particle-properties’ of any agent and its relation to the organization of water e.g. NaCl that gets dissociated into Na^+^ and Cl^–^ has about the same osmotic effect on cells at 0.5 M as sorbitol at 1 M (Blomberg and Adler 1992). However, this rule of thumb applies to lower concentrations, and more agent-specific effects on the water activity appear at higher concentrations. Besides the osmotic effect there might be additional effects from specific properties of the stress-agent. For example, addition of NaCl increases the ionic strength of the solution, but might also have more specific biological effects e.g. Na^+^ is generally more toxic than K^+^ at equimolar concentrations/same ionic strength (Blomberg and Adler 1992). Na^+^ is therefore transported out of the cell to keep intracellular levels low via active sodium transporters like Ena1 and Nha1 (Hohmann 2002). In contrast to NaCl, sorbitol has the benefit of being exclusively ’osmotic’ because of its non-ionic nature. This polyol is generally regarded to be poorly assimilated by *S. cerevisiae* which makes it a potentially metabolically inert stress-agent. However, *S. cerevisiae* strains can eventually grow on sorbitol but for most strains after an extensively long lag-phase (Sarthy *et al*.^1994^). Furthermore, mutations in the transcriptional corepressor components Tup1 or Cyc8, which leads to de-repression of the sorbitol dehydrogenase SOR2 and sorbitol transporters HXT15/17, result in the ability to assimilate sorbitol (Chujo *et al*. 2015, Tanaka *et al*. 2020). Thus, both the growth regime and the genetic background can influence the studied response to osmotic stress imposed by high sorbitol concentrations. In addition, a complicating factor is that sorbitol is a polyol and is thus generally benign to the cellular machinery and could if added externally as a stress-agent potentially accumulate intracellularly to act as an osmolyte. In fact, sorbitol plays the role of an osmolyte in some organism (Yancey 2005), and to some extent it can also replace glycerol's osmo-protective role in yeast (Shen *et al*. 1999).

Despite these fundamental differences between NaCl and sorbitol as osmotic stress-agents, few are the systematic studies where strict comparisons have been made between their cellular responses. In a recent study under standardized conditions the potential of large numbers of yeast species/strains were subjected to phenotypic screens to a wide range of stresses, among them screens for osmotolerance (sugars/sorbitol) and for halotolerance (NaCl/KCl) (Mukherjee *et al*. 2017). The authors reported that many of the species that tolerate high sugars/sorbitol also showed tolerance to high salt, which indicates common molecular mechanism to withstand sugar/sorbitol and salt stress. However, there were also species that clearly differed in their tolerance to salt and sorbitol, where for example *S. pombe* and strains from the *Zygosaccharomyces* genus were identified as osmotolerant but found to be sensitive to salts. Other studies also indicate a substantial overlap in the gene expression response to either NaCl or sorbitol (to roughly similar external osmolarity), suggesting that most of the changes in gene expression are in response to the osmotic change (Causton *et al*. 2001, Hirasawa *et al*. 2006). In summary, studies on osmoregulation using either NaCl or sorbitol as stress agent display many similarities, however, studies should ideally use both these stress-agents at least to confirm that the most important osmo-phenomena under study are ‘osmotic’ and not ‘specific’ for the stress-agent in use.

**Figure 1.**
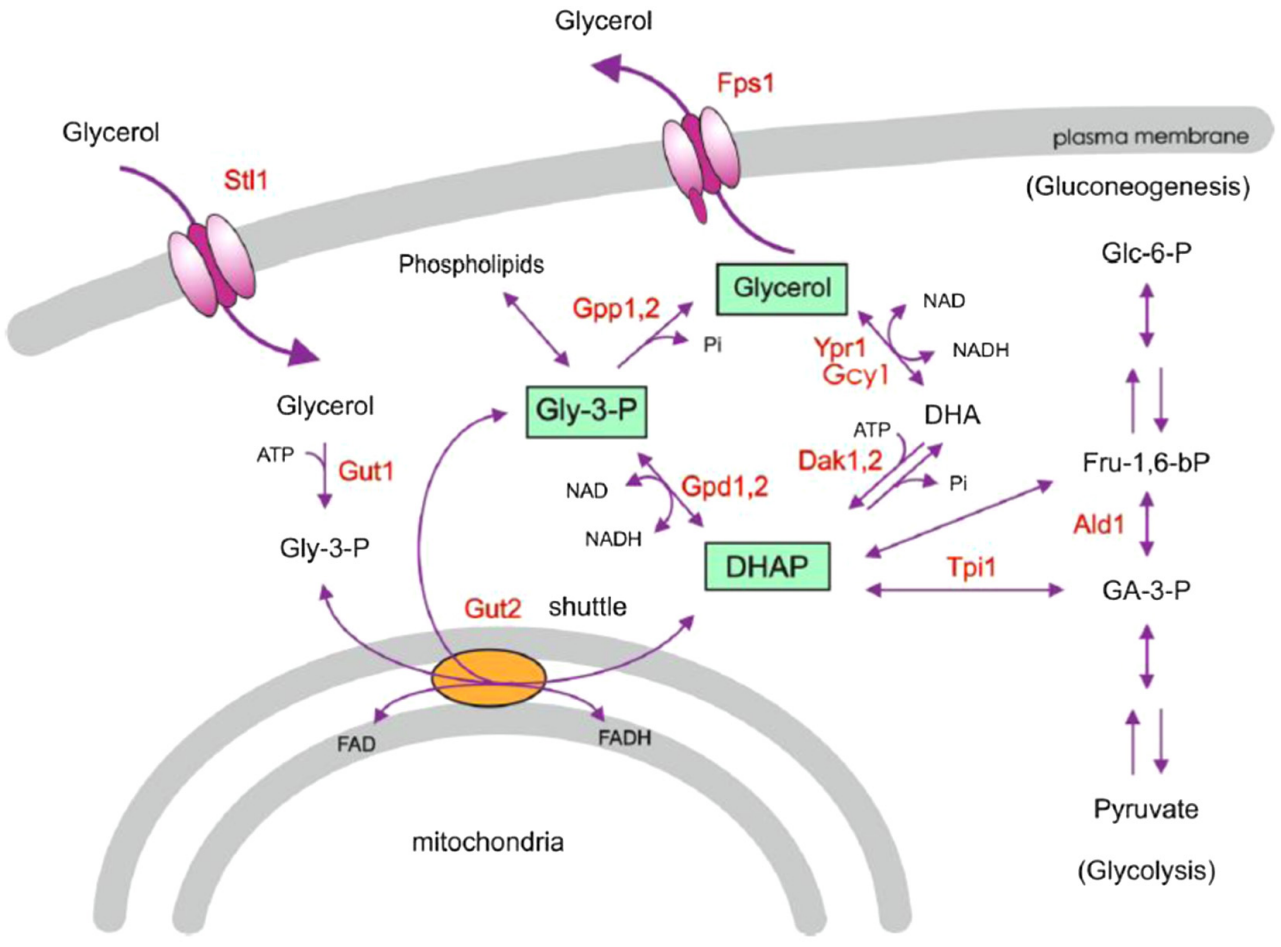
Glycerol metabolism in yeast. Glycerol is produced from the glycolytic intermediate dihydroxyacetone phosphate DHAP. The formation of glycerol is catalyzed by the NAD-dependent glycerol-3-phosphate dehydrogenase, Gpd1 and Gpd2, and glycerol-3-phosphatase, Gpp1 and Gpp2. In addition, there is an alternative pathway for glycerol production via dihydroxyacetone and the enzymes Dak1/Dak2 and Gcy1/Ypr1, however, this pathway does not seem to play a role in glycerol production under osmostress in yeast but have been reported important in this process in the fungi *Aspergillus nidulans* (de vries 2003). Intracellular accumulation of glycerol is regulated by the aquaglyceroporin Fps1. Glycerol is assimilated by active uptake via the glycerol/H^+^-symporter Stl1, phosphorylated by the glycerol kinase Gut1 and oxidised by the FAD-dependent glycerol-3-phosphate dehydrogenase Gut2 in the mitochondrial membrane to DHAP, which then enters glycolysis/gluconeogenesis. This figure is from (Hohmann 2015) and is published with permission from Springer Nature.

Microorganisms counteract dehydration through intracellular accumulation of one or more specific solutes that are harmless with respect to the intracellular machinery, which are called compatible solutes (Brown and Simpson 1972) or osmolytes (Yancey *et al*. 1982). Accumulation of intracellular osmolytes leads to the re-entry of water back into the cell solely through osmotic means (water cannot be pumped). The main osmolyte that accumulates in yeast cells is glycerol (Blomberg and Adler 1989, Blomberg and Adler 1992, Hohmann 2015). Glycerol is produced in a two-step pathway from the glycolytic intermediate dihydroxyacetone phosphate (DHAP), where the first step is catalyzed by the cytosolic NAD-dependent glycerol-3-phosphate dehydrogenase (Gpd1 and Gpd2) and the second step by glycerol-3-phosphatase (Gpp1 and Gpp2) (Fig. 1). This is the exclusive route for glycerol production in yeast since deletion of either isogene-pair, *gpd1∆gpd2∆* (Ansell *et al*. 1997) or *gpp1∆gpp2∆* (Norbeck *et al*. 1996), results in complete abolishment of glycerol production. A second pathway for glycerol production using dihydroxyacetone (DHA) as an intermediate (Fig. 1) has been experimentally verified in the filamentous fungi *Aspergillus nidulans* to occur via a NADPH-dependent glycerol dehydrogenase (de Vries *et al*. 2003), however, this pathway does not seem to be important for glycerol production in *S. cerevisiae* during osmostress. The accumulated glycerol is exported via the aquaglyceroporin Fps1 if the osmotic conditions changes and cells experience hypoosmotic stress (Tamas *et al*. 1999, Hohmann 2002).

Glycerol is not only produced by yeast, but it can also be taken up from the external media via the H^+^-coupled active transporter Stl1, both during growth on glycerol as the sole carbon and energy source as well as during osmostress (Ferreira *et al*. 2005). The mutation/deletion of either glycerol kinase *GUT1* or the mitochondrial FAD-dependent glycerol 3-phosphate dehydrogenase *GUT2* result in complete abolishment of growth of *S. cerevisiae* on glycerol as the sole carbon and energy source (Sprague and Cronan 1977, Swinnen *et al*. 2013), strongly suggesting that glycerol is exclusively utilized via the G3P pathway during aerobic conditions (Fig. 1). The DHA-pathway has also been proposed to be a glycerol utilization route, however, the involvement of the putative glycerol dehydrogenases/aldo-keto reductases Ypr1 and Gcy1 in glycerol utilization (Fig. 1) is debated, and conflicting results have been presented for their involvement (Jung *et al*. 2012, Klein *et al*. 2017, Asskamp *et al*.^2019^). In addition, two of the enzymes in this alternative glycerol utilization-pathway, Dak1 and Dak2, are suggested to be involved in stress-imposed detoxification of DHA since this intermediate is toxic and produces advanced glycation end-products on proteins (Molin *et al*.^2003^).

For redox-balancing reasons glycerol is not only produced under osmotic stress in yeast but also as a byproduct in normal non-stress media during anaerobic conditions (Nordstrom 1966, Blomberg and Adler 1992, Hohmann 2015). The reason for this production is that glycerol serves as the major redox sink for reducing equivalents produced in biosynthetic pathways during growth without oxygen. Under aerobic conditions, the intermediate glycerol 3-phosphate is part a redox-shuttle to optimize the cytoplasmic NAD^+^/NADH ratio via the mitochondrial Gut2 and respiration (Larsson *et al*. 1998).

The simplistic view that these enzymes in glycerol production/accumulation would be the only changes in cell during the response to dehydration was early on challenged and the response shown to be much more complex, as revealed by the rapid and very diverse and drastic changes in protein expression (Blomberg 1995) and gene expression (Gasch *et al*. 2000, Causton *et al*. 2001) even during short times of acclimation to hyperosmotic conditions. However, many of these changes in gene expression appear not to be individually important, since single gene deletions of these expression responders do not generally result in osmo-sensitivity (Warringer *et al*. 2003). In summary, despite extensive changes in gene/protein expression in response to osmotic challenge, enzymes involved in glycerol production and accumulation is still regarded to be the key-factor in the cellular response to osmotic stress.

Cells respond to external stimuli by activation of well-conserved signaling pathways, where the high osmolarity glycerol (HOG) pathway is the most prominent (Saito and Posas 2012, Brewster and Gustin 2014). The signal is most often transmitted within the cell by reversible protein phosphorylation where the addition and removal of the phosphate moiety can modulate proteins' activity, localization, and interactions with other proteins. For the cell to adequately respond to specific situations, signal propagation and integration mechanisms need to be intricately fine-tuned. A comprehensive mathematical model of the cellular response of yeast to hyperosmotic shock has been developed and validated (Klipp *et al*. 2005). The model integrates biochemical reactions, sensor stimulation, the mitogen-activated protein kinase cascade, and activation of gene expression with a thermodynamic description of volume regulation and osmotic pressure. Importantly, model simulations agreed with experimental results obtained under hyperosmotic conditions and the model was thus shown to be predictive. This model has recently been extended to include single-cell dynamics during growth under osmotic stress (Altenburg *et al*. 2019). It was found that single-cell growth rate and final cell-size are primarily governed by osmolyte uptake and consumption. These important steps in modeling cellular responses to stress are truly landmark events in the strive to transform biology from a descriptive science to a predictive science.

Hyperosmotic stress has been an exceptionally fruitful line of research for understanding cellular responses to environmental challenges, and many aspects of the stress-activated machinery have been revealed with great molecular resolution and several excellent reviews concerning molecular aspects have been presented over the last two decades (Hohmann 2002, Hohmann *et al*.^2007^, Saito and Posas 2012; Brewster and Gustin 2014, Hohmann 2015). Stefan Hohmann's seminal review from 2002 stands out to be extremely informative and comprehensive, providing inspiration and background in various aspects of the response and still attracting a lot of attention with now in total > 1800 citations (Google scholar, January 2022). I will in this review not focus too much on earlier results already presented in these comprehensive earlier reviews, but instead reflect on more recent findings that expand our knowledge on already well-studied osmoregulatory mechanisms or add totally new avenues in our understanding of osmoregulation. It is fascinating that such a simple system—salt/sorbitol and yeast—has developed into such an enormously potent model system unravelling important fundamental aspects in biology.

## SENSORS THAT RESPOND TO OSMOTIC DEHYDRATION

### Protein-mediated osmo-sensing

**Figure 2.**
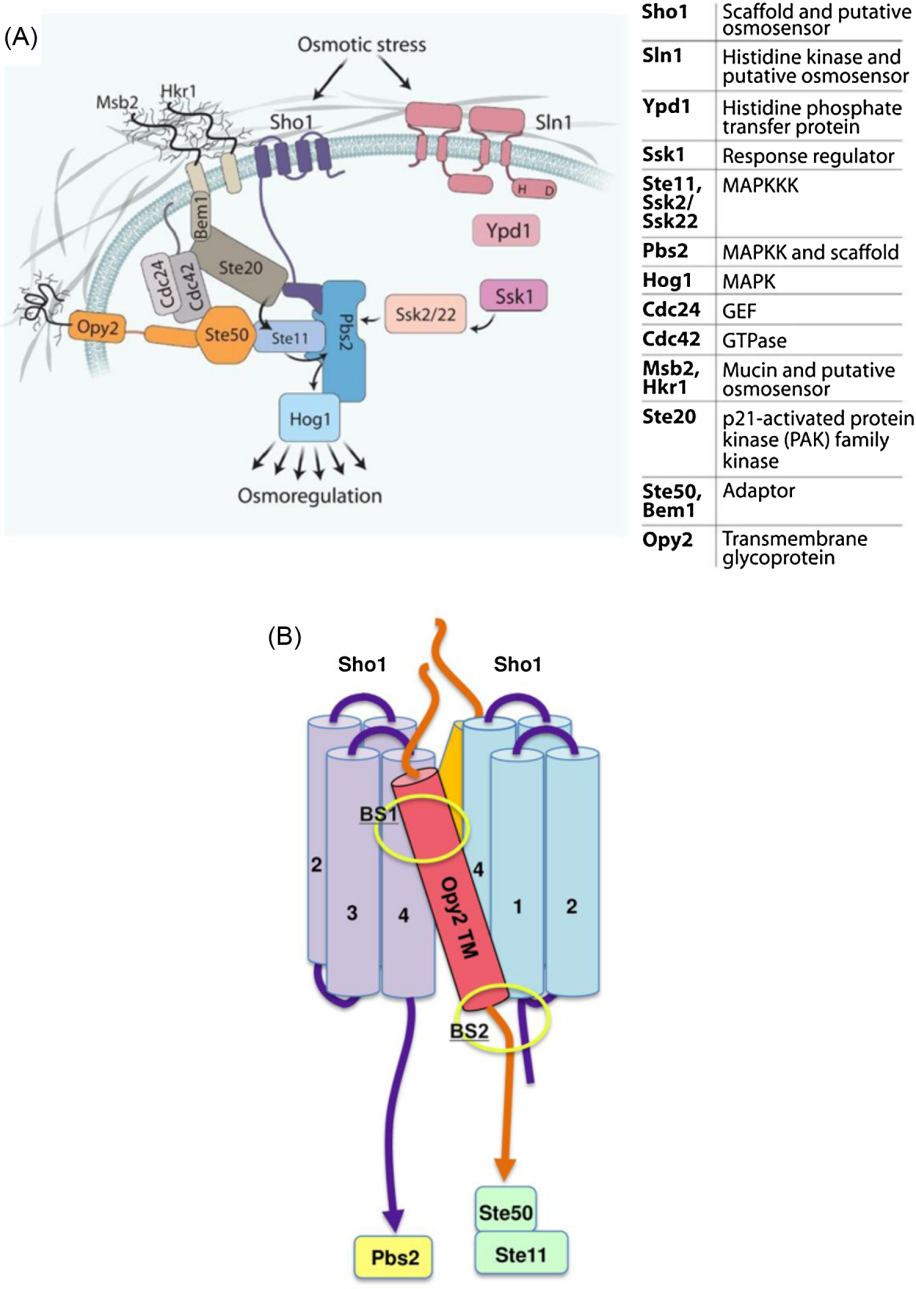
A) The model of osmo-sensing and osmo-signaling of the HOG pathway in *S. cerevisiae*. For the Sln1 branch, osmotic stress releases Sln1-dependent inhibition of Ssk2/22 to activate the pathway. For the Sho1 branch, activation requires the membrane-embedded mucin proteoglycans Msb2 or Hkr1 to interact with Sho1 and Ste20 in a complex with the MAPK components. Opy2 is a transmembrane glycoprotein that serves as an anchor for Ste50. Cdc24 and Cdc42 are the cytosolic guanosine triphosphatase (GTPase) and guanine nucleotide exchange factor (GEF) that activate Ste20.This figure is from (Brewster and Gustin 2014) and is published with permission from the American Association for the Advancement of Science. B) Schematic model of the transmembrane interactions between a Sho1 dimer and two Opy2 proteins. Transmembrane (TM) domains of Sho1 and Opy2 are indicated as light purple/blue and red cylinders, respectively. This figure is from (Takayama *et al*. 2019).

A central question in yeast osmoregulation is what sensing mechanisms act upstream of glycerol production/accumulation and other osmo-induced adjustments. The first putative osmosensor identified in yeast was Sln1, a protein with sequence homology to prokaryotic two-component signaling systems (Saito and Posas 2012, Brewster and Gustin 2014). The design of these prokaryotic stress pathways involves a ‘sensor’ protein that transfers high-energy phosphate through a histidine phosphorelay to a ‘receiver’ protein and subsequent down-stream signaling. Sln1 is localized within the cell membrane by two transmembrane (TM) domains (Fig. 2A). Mutagenesis studies of Sln2 has shown that the osmotic regulation depends on the N-terminal extracellular domain and one of the associated TM domains, which together are believed to sense macromolecular crowding through both membrane-associated and cell wall–associated components. Supporting this is the fact that removal of the cell wall, gene deletion of the cell wall protein Ccw12, or permeabilization of the cell membrane with nystatin, inactivate Sln1 such that it is not responsive to modulation by extracellular osmolytes (Saito and Posas 2012).

The next osmo-sensor gene identified encoded the integral membrane protein Sho1 that contains four TM domains and a single cytosolic SH3 domain that binds to Pbs2 (Fig. 2A). It has been shown that Sho1 is a functional osmo-sensor by itself (Tatebayashi *et al*. 2015). The four TM domains are responsible for Sho1 oligomerization, however, the oligomer structure was not altered by external high osmolarity. Interestingly, Sho1 trimerizes at the TM2/TM3 interface and dimerizes at the TM1/TM4 interface. It is yet not clear if this repetitive ‘dimers-of-trimers’ architecture is part of Sho1’s osmo-sensing capacity. Sho1 is now recognized as more than just an osmo-sensor since it also serves as a critical adaptor protein forming membrane-associated complexes that take part in sensing/signaling (Brewster and Gustin 2014).

Furthermore, Msb2 and Hkr1 are additional osmo-sensors that are heavily glycosylated mucin-like transmembrane proteoglycans. They both physically interact with the chitin and glucan network of the cell wall and this interaction is the potential base for osmo-sensing, where both have the capacity to activate HOG pathway signaling through Sho1 (Fig. 2A). The highly glycosylated part of Msb2p is necessary for its activity (Yang *et al*. 2009). The model is that dehydration pulls the cell membrane away from the cell wall which will be physically sensed by Msb2 and Hkr1 and subsequently stimulate the HOG pathway. Interestingly, Msb2 and Hkr1 mechanisms of interaction with the HOG pathway are distinct; activated Msb2 interacts with cytosolic Bem1 and in that way recruits the Ste20 or Cla4 enabling their activation of Ste11, while the Hkr1 activation of the HOG pathway does not require Bem1 (Tanaka *et al*. 2014). It has been shown that Msb2 also binds to the Opy2 protein (Yamamoto, Tatebayashi and Saito 2016). Membrane localization of Ste50 is an important function of Opy2, since localization of Ste50 to the membrane by adding a C-terminal prenylation site suppresses the Hog1 activation defect in the *opy2∆* strain (Tatebayashi *et al*. 2007).

It was recently shown that the single TM-domain of Opy2 interacts with the TM-domain of Sho1 (Takayama *et al*. 2019). The cytoplasmic C-terminus of Opy2 carries functionally distinct binding sites for the adaptor Ste50 that is needed for Ste11-Pbs2 signaling (Fig. 2A). This interaction between the TM-domains of Sho1 and Opy2 places their respective cytoplasmic binding partners Pbs2 and Ste11 in proximity and promotes the interaction between Pbs2 and Ste11. However, it was shown that the binding between Opy2 and Sho1 was not influenced by high osmolarity and thus binding is possibly not part of the osmo-sensing activity but might rather be an essential mechanism for the function of the osmo-sensor complex. Instead, it was hypothesized that Sho1, with its four tightly packed TM-domains, forms two separate Sho1 interfaces for the binding of Opy2 (Fig. 2B). If the Opy2 extracellular and cytoplasmic ends of the TM-domain are fixed to different molecules of Sho1, the tilt angle (relative to the membrane plane) of the Opy2 TM-domain might sense the structural distortion of the Sho1 oligomers as a result of osmotic stress. An interesting working-model is proposed in which a small change in the Opy2 tilt angle will by a ‘leverage mechanism’ that bring Ste11 and Pbs2 together and initiate signaling.

### Membrane-mediated osmo-sensing

It has become clear that the plasma membrane, in addition to acting as a protective barrier around the cell, takes an active part in a wide range of cellular processes including endocytosis, secretion, nutrient uptake, ion homeostasis, signal transduction, morphogenesis, and cell wall synthesis (Lanze *et al*. 2020). Apparently, the plasma membrane coordinates these diverse processes through distinct membrane domains that orchestrate different activities. The semipermeable property of the plasma membrane leads to a higher osmotic pressure at the cell interior than at the external environment due to impermeant intracellular metabolites. This osmotic gradient is an important mechanical force acting on cell membranes that exerts a lateral tension that stretches the lipid bilayer and produces a reduction in membrane thickness (Cohen 2018). Changes in bilayer thickness and alterations of membrane curvature are now recognized as critical modulators of the function of some membrane proteins.

The presence of unique functional domains in the plasma membrane has in particular been well studied in the *S. cerevisiae*, where at least five non-overlapping domains have been described based on their inclusion of different reporter proteins: MCC, stands for membrane compartment of the arginine permease Can1, MCP, stands for membrane compartment of H^+^-ATPase Pma1, MCT, stands for membrane compartment of TORC2 kinase, MCL, stands for membrane compartment of sterol transporter Ltc3/4, and MCW, stands for membrane compartment of cell wall mechanosensor Wsc1 (Athanasopoulos *et al*. 2019). The MCC domain correspond to earlier observed inward furrows of the plasma membrane that was reported decades ago by the use of freeze-etch electron microscopy (Moor and Muhlethaler 1963). These domains are about 200 to 300 nm long, 50 nm wide, and 50 nm deep in *S. cerevisiae* under standard growth conditions. These furrows are stabilized by several proteins, forming a complex termed the eisosome, and there are typically about 50 MCC/eisosome domains per yeast cell distributed throughout the plasma membrane. The formation of the MCC/eisosomes is promoted by two homologous proteins, Pil1 and Lsp1, which contain Bin-amphiphysin-Rvs domains that bind the cytoplasmic surface of the plasma membrane and form long filaments that shape the furrows (Lanze *et al*. 2020). In addition, sphingolipids and sterols are known to be tightly packed together in cell membranes to form domains called lipid rafts. It has been reported that inhibition of the *de novo* sphingolipid synthesis pathway results in activation of the osmo-sensitive HOG pathway (Tanigawa *et al*. 2012; Yamaguchi *et al*. 2018). In this context it is interesting that both Sho1 and Sln1 copurify with detergent-resistant lipid rafts, with osmotic stress differentially influencing their localization: Sln1 decreases and Sho1 increases in these rafts (Tanigawa *et al*. 2012). These observations imply that yeast cells might sense osmotic stress via the structural and/or physical properties of lipid rafts. It was recently proposed that membrane thickening/thinning in response to osmotic changes leads to tilting of the TM domains of Sln1 and Sho1 leading to signaling via the HOG pathway (Cohen 2018).

It has been reported that the target of rapamycin complex 2 (TORC2) acts like a regulator of cell surface area and plasma membrane tension and was proposed to be part of a homeostatic feedback loop that maintains tension in the plasma membrane (Berchtold *et al*. 2012). It was shown that the proteins Slm1 and Slm2 take part in TORC2 signaling, specifically upstream of TORC2 in the perception of an increase in plasma membrane tension. Interestingly, Slm1 was scored in an earlier genome-wide study as an osmosensitive knock-out strain, but then under the systematic name YNL047c since it was at the time not functionally characterized (Warringer *et al*. 2003). In a recent study, modulators of plasma membrane tension i.e. palmitoylcarnitine (PalmC) as well as hyperosmotic shock, were used for mechanistic studies of this TORC2/Slm-dependent sensing (Riggi *et al*. 2018). It was found that neither treatment affected Slm1 localization, but instead both induced the phase separation of phosphatidylinositol-4,5-bisphosphate (PtdIns(4,5)P2) into pronounced plasma membrane invaginations. Interestingly, cells lacking some of the proposed osmo-sensing components of the HOG pathway (e.g. *slni1∆pbs2∆* or *hkr1∆msb2*∆) or of the cell wall integrity (CWI) pathway (e.g. *wsc1∆mid2∆)* retained the ability to regulate TORC2 activity following osmotic shocks, indicating that the osmo-sensor(s) upstream of TORC2 must be HOG/CWI-independent.

It has earlier been shown that TORC2 is recruited to the plasma membrane via the pleckstrin homology domain of its Avo1 subunit which binds PtdIns(4,5)P2 (Berchtold and Walther 2009). Interestingly, decreased plasma membrane tension leads to that plasma membrane domains are enriched in PtdIns(4,5)P2. In contrast, these PtdIns(4,5)P2-enriched structures (PESs) quickly disassemble on plasma membrane stretching induced by a hypoosmotic shock, thus supporting the idea that a decreased plasma membrane tension as under a hyperosmotic chock constitutes the primary cause of formation of these PESs. It was concluded that hypo- and hyperosmotic shocks are sensed upstream of TORC2 by distinct molecular mechanisms: i) sensing of increased plasma membrane tension (hypoosmotic shock) involves the translocation of Slm proteins from MCC/eisosomes to the MCT domains where they activate TORC2; and ii) decreased plasma membrane tension (hyperosmotic shock) triggers a spontaneous PtdIns(4,5)P2 phase separation into differentially ordered sub-domains that inactivate TORC2 (Riggi *et al*. 2018). Thus, the plasma membrane *per se* could act as one of the primary sensors that respond to osmo-induced changes in membrane properties that activates down-stream signaling routes.

### Cytoplasmic ‘crowding’ osmo-sensing

It has been proposed that macro-molecular crowding could provide scope for mechanisms of intracellular osmo-sensing (Hohmann 2015). A rather moderate drop of the intracellular water concentration leads to macro molecular crowding, reduced free diffusion and slow-down of the molecular processes (Mika and Poolman 2011). In this context it is interesting that the protein family LEA (late embryogenesis abundant) from the plant *Arabidopsis thaliana* are intrinsically disordered proteins that exhibit a reversible disorder-to-order/folded transition in response to increased osmolarity *in vitro* (Cuevas-Velazquez *et al*. 2016). The LEA proteins are rather small (≈ 160 amino acids), show high hydrophilicity, high content of small amino acids (usually > 6% glycine), and absence/deficit of hydrophobic residues, in analogy with a large family of proteins called hydrophilins. Yeast encodes 12 hydrophilins, among others Hsp12, Stf2 and Sip18 (all three induced by osmotic stress; (Garay-Arroyo *et al*. 2000). It is also interesting that several of the components in the HOG-pathway contains intrinsically disordered shorter regions, that when being deleted, result in drastic changes in the *in vivo* performance of the HOG-components (Strome *et al*. 2018).

The plant LEA protein was fused between a FRET-compatible pair of fluorophores, building a genetically encoded fluorescent biosensor called SED1 that would record osmolarity- and crowding-dependent conformational changes inside living yeast cells (Cuevas-Velazquez *et al*. 2021). The construct was expressed in the yeast cytoplasm and exhibited a significant NaCl-concentration-dependent increase *in vivo* in the acceptor-to-donor emission ratio. Interestingly, it was shown that the sensitivity of the osmolarity-response was not just a general property of intrinsically disordered peptides but was a specific property of the LEA sequence. Even if SED1 is an ectopically expressed reporter construct, its response in yeast clearly exemplifies that proteins can quantitatively respond to the intracellular osmolarity/crowding and could evolve to become cytoplasmic osmo-sensors. If this kind of intracellular osmo-sensing protein endogenously exist in yeast is currently not known, but certainly deserves attention in the future.

## DIFFERENT SIGNALING ROUTES ARE ACTIVATED DURING HYPEROSMOTIC STRESS

### HOG-pathway signaling

Changes in the environment lead to rapid adjustment of cellular physiology orchestrated by activating signaling networks that involve multiple component phosphorylations, with kinases often being positive effectors while phosphatases are antagonists. Both the addition and removal of the phosphate moiety can modulate protein functionality, activity, localization, and protein-protein interactions. The initial discovery and the subsequent science behind the characterization of what have become known as the HOG-pathway have been vividly studied for three decades (Saito and Posas 2012, Brewster and Gustin 2014). Screening of mutagenized yeast for reduced growth and reduced glycerol accumulation during hyperosmotic conditions led to the identification of an unknown MAPK (mitogen activated protein kinase) cascade that is key in eliciting a proper stress response (Brewster *et al*. 1993). Four complementation groups were identified, of which two turned out to be mutated in genes encoding protein kinases, *PBS2* and *HOG1*. Mutations that inactivate the HOG pathway make yeast highly sensitive to hyperosmotic stress while mutations causing uncontrolled HOG pathway activity are lethal. It turns out that the HOG pathway contains two redundant signaling/sensing routes upstream of Hog1 and Pbs2, the Sln1-branch and the Sho1-branch (Fig. 2A; see above). Much of the molecular mechanics of this signaling pathway was unraveled by Saito and Posas by applying elegant genetics (Saito and Posas 2012).

In short, Sln1 is a negative regulator of the HOG pathway branch with the Sln1 histidine kinase being active under normal growth conditions. The Sln1 activity is the result of autophosphorylation which leads to transfer of the phosphate group, via the phosphotransfer protein Ypd1, and activation of the response regulator protein Ssk1. Upon hyperosmotic shock Sln1 is inactivated and Ssk1 becomes dephosphorylated, which then makes it interact with and activate the two MAPKKK protein kinases, Ssk2, and Ssk22 via auto-phosphorylation. Activated Ssk2/22 activate the MAPKK Pbs2 by phosphorylation which in turn phosphorylates MAPK Hog1 on two adjacent phosphorylation sites (Thr174 and Tyr176), leading to its activation. One of the most prominent features of phosphorylated Hog1 is its accumulation in the nucleus where it plays a vital role in activating transcription of a wide array of stress-genes (see below), however, it also has some cytoplasmic targets.

The Sho1-branch has several putative sensors (see above) and the signaling is initiated by activation of the membrane-localized scaffold protein Sho1 which then interacts with different components of the pathway. The Sho1 activation leads to membrane-localization of the G-protein Cdc42, and the protein kinases Ste20 and Cla4. The Cds42-Ste20-Cla4 complex phosphorylates and activates the MAPKKK Ste11. Via interaction with Sho1, Pbs2 is then recruited to the membrane in its additional role as a scaffolding protein, and Ste11 is recruited to the membrane by its association partner Ste50 by interaction with Cdc42, Sho1 and another membrane protein, Opy2. The activated Ste11 in turn phosphorylates and activates Pbs2, which phosphorylates and activates Hog1. Protein phosphatases like the type 2C serine/threonine phosphatase Ptc1 and the phospho-tyrosine phosphatases Ptp2 and Ptp3 act as negative regulators/modulators of the HOG pathway (Brewster and Gustin 2014).

The Hog1 kinase is important for cell survival under hyperosmotic conditions where it plays multiple roles in gene expression, metabolic regulation, signal fidelity and cell cycle regulation (Brewster and Gustin 2014, Hohmann 2015). Several Hog1-dependent targets have been described in the literature. Given that the Hog1 dependency of many of these sites has been established using *in vitro* kinase assays one can question if they are genuine *in vivo* substrates of Hog1. The phosphorylation dynamics of the hyperosmotic response *in vivo* have been studied by the use of phosphoproteomics, reporting the changes to be complex and involving many kinases and phosphatases (Soufi *et al*. 2009, Kanshin *et al*. 2015). Many direct and indirect targets of Hog1 have been suggested, however, in most cases it is hard to decipher which functions are directly controlled and which are indirectly controlled by Hog1.

To distinguish between direct and indirect Hog1 targets, the *S. cerevisiae* phosphorylome was studied with quantitative mass spectrometry (MS) after 5 minutes of osmostress in the wild type comparing that to a yeast strain with a point mutation in the endogenous Hog1 locus (i.e. Hog1as) that renders the Hog1 protein sensitive to inhibition by an ATP analog (Romanov *et al*. 2017). In addition, the phosphorylated targets were confirmed to have direct interaction with Hog1 with a protein-protein proximity assay designed to capture transient interactions between kinases and their substrates. In this way it was established that in total 40 proteins are direct targets of Hog1, of which 32 had not been described previously e.g. Bck1, Hal5, Ppz2 and Tsl1. It was also examined how a deletion of the known effector protein kinase target of Hog1, Rck2, influences the osmotic stress–induced phosphorylation. It was found that the Rck2-dependent phosphoproteome to be complex involving > 300 phosphorylation sites and seemed to involve a large portion of the identified indirect targets of Hog1. Thus, Rck2 is a major effector kinase of Hog1 influencing many Hog1 secondary targets representing a wide array of cellular processes. It is also proposed that Rck2 is a central hub of a phosphorylation network affecting the phosphorylation of > 16 kinases. The central position of Rck2 in the regulatory osmotic stress-signaling network is also confirmed by a combined phosphoproteomics and computational approach (MacGilvray *et al*. 2018). In summary, these studies emphasize the surprisingly wide set of cellular functions, both direct and indirect, that show an Hog1-mediated phosphorylation response during hyperosmotic stress (de Nadal and Posas 2022).

### TORC2-Ypk1 signaling

A link between TORC2-Ypk1 signaling and hyperosmotic regulation was established when it was shown that Gpd1 under normal growth conditions is phosphorylated by the protein kinase Ypk1, which activity is downregulated by osmotic stress (Lee *et al*. 2012). This discovery was inspired by a large-scale analysis of protein kinase phosphorylation site motifs in yeast (Mok *et al*. 2010). Based on this motif analysis the substrates for a substantial portion of the protein kinases were predicted with a combined bioinformatics and peptide library screening approach. This led to that both Gpd1 and Gpd2 were identified as candidate substrates for the AMP-activated protein kinase Snf1, however, it was experimentally established that only Gpd2 was phosphorylated by Snf1 (Lee *et al*. 2012). Instead, Gpd1 phosphorylation was identified to serine-24 (sequence R-K-R-S-S-pS in Gpd1) that overlapped with the protein kinase Ypk1 phosphorylation motif (R-X-R-X-X-pS). Subsequently, the phosphorylation of Gpd1 by Ypk1 at this site was confirmed both *in vitro* and *in vivo* (Lee *et al*. 2012).

**Figure 3.**
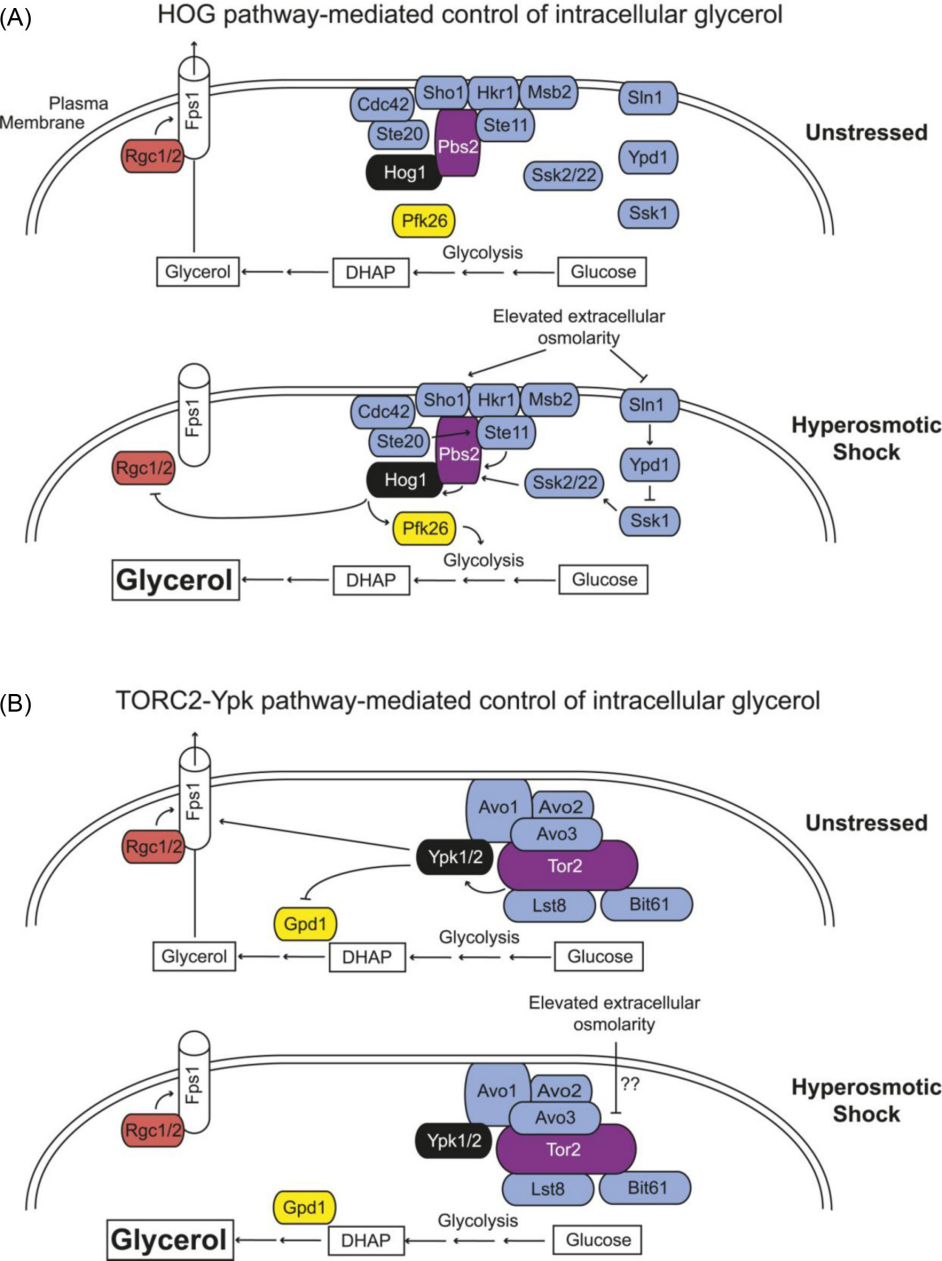
*Saccharomyces cerevisiae* has two independent sensing and signaling systems to rapidly increase intracellular glycerol. The figure depicts a schematic representation of the involved components for each system. These processes act synergistically to elevate the intracellular glycerol concentration to counterbalance the external osmotic stress. **(A)** HOG pathway-mediated control of intracellular glycerol. In unstressed conditions (upper panel) Hog1 is inactive and glycerol is generated as a minor side product of glycolysis to regulate the cytoplasmic NADH/NAD^+^ imbalance during fermentative growth. Produced glycerol escapes the cell through the Fps1 channel that is maintained in its open state by the bound regulators Rgc1 and Rgc2. Upon hyperosmotic stress (bottom panel) components coupled to the Sho1 and Sln1 osmosensors lead to Hog1 phosphorylation and activation. Activated Hog1 increases intracellular glycerol via both phosphorylation of Pkf26 in the cytosol (leading to enhanced glycolytic flux) and phosphorylation of Rgc1 and Rgc2 preventing glycerol efflux through Fps1. Activated Hog1 also enters the nucleus (not depicted in the figure) where it transcriptionally upregulates *GPD1* and several other osmostress genes. **(B)** TORC2-Ypk-mediated control of intracellular glycerol. In unstressed conditions (upper panel) active TORC2-Ypk1 keeps intracellular glycerol level low by enzymatic inhibition via phosphorylation of Gpd1 and by phosphorylation stimulating the open state of Fps1. Upon hyperosmotic stress (bottom panel) the TORC2-dependent phosphorylation of Ypk1 is rapidly down-regulated that leads to that the inhibition of Gpd1 is alleviated, thereby increasing glycerol production. Concomitantly, loss of Ypk1-mediated phosphorylation closes the Fps1 channel, promoting glycerol accumulation. This figure is from (Muir *et al*. 2015).

Ypk1 is a well-established TORC2 substrate (Roelants *et al*. 2011; Muir *et al*. 2014). There are two types of target of rapamycin (TOR) complexes in *S. cerevisiae*: TORC1 and TORC2 that differ in their regulation and downstream effectors (Tafur, Kefauver and Loewith 2020). Despite its name, the TOR complex 2 is rapamycin insensitive; it consists of Tor2 (a rather large, ≈2700 amino acids, phosphatidylinositol 3-kinase), Avo1, Avo2, Avo3 (the Avo-proteins appear to bind to phosphoinositide-(4,5)-bisphosphate), Bit61 and Lst8 (contains a WD repeat)(Fig. 3B). As shown by various protein-protein interaction assays, the Avo2 subunit of TORC2 also associates with Slm1 and Slm2. It has been shown that Slm1 and Slm2 are required for TORC2-mediated phosphorylation of Ypk1, possibly via binding to and delivering Ypk1 to TORC2 (Berchtold *et al*. 2012). However, alternative mechanisms for the Ypk1-TORC2 interaction have been proposed, where instead Avo1 binds Ypk1 and deliver it to the active site of Tor2 (Liao and Chen 2012). Ypk1 is also activated by phosphorylation on the conserved threonine residue Thr504 in its activation loop by the eisosome-associated protein kinase Pkh1, where the phosphatase PP2A has been proposed to be responsible for its dephosphorylation (Roelants *et al*. 2011). Hyperosmotic conditions dramatically prevent TORC2-mediated phosphorylation of Ypk1 at Thr662, and thus prevents Ypk1 from phosphorylating its targets Gpd1 and Fps1 (Muir *et al*. 2015). This deactivation of Ypk1 is a very rapid response and happens within a minute, however, the response is transient and is back at pre-stress level by a bit more than an hour. It was recently reported that four newly identified C-terminal sites are also important for Ypk1 activity, stability, and biological function (Leskoske *et al*. 2017).

### PP2A phosphatase signaling

Hog1 has a central role in hyperosmotic stress, however, it regulates only one third of the stress-dependent phosphorylome (Romanov *et al*. 2017), indicating that other mechanisms, like TORC2-Ypk1 and possibly others, regulate the remaining stress-induced phosphorylation events. Most studies propose a rather passive role of phosphatases in terminating kinase signaling cascade, and in that way secondarily affecting the phosphorylation state of target proteins. However, in recent years it has been shown that many stress-induced phosphorylation sites are affected by a phosphatase in a primary manner, rendering the phosphatase a main downstream signaling effector of the stress response. By combining isotopic labeling and quantitative mass-spectrometry phosphoproteomics the mechanistic underpinning of some of the Hog1-independent phosphorylation events during hyperosmotic stress were studied, mainly focusing on the importance of the phosphatase PP2A (Hollenstein *et al*. 2021). PP2A is a multi-protein complex, consisting of a catalytic, a scaffolding, and a regulatory subunit, where substrate specificity is defined by the two regulatory subunits Cdc55 and Rts1. These two regulatory subunits have previously been linked to the hyperosmotic stress response (Evangelista *et al*. 1996; Reiter *et al*. 2013). It was found that 52 and 25% of phosphorylation sites with an increased abundance upon hyperosmotic stress treatment, also showed an increase in *cdc55Δ* and *rts1Δ* cells, respectively (Hollenstein *et al*. 2021). Thus, there were a great overlap in targets commonly affected by hyperosmotic stress and those affected by the PP2A activity. Interestingly, it was reported that stress-induced sites that are independent of Hog1 were only affected by the deletion of *CDC55* and not *RTS1*. PP2A-Rts1 was instead a specific branch of Hog1 signaling. Thus, PP2A seems to be involved in different signaling branches during hyperosmotic conditions, depending on its bound regulatory subunit. The hyperosmotic stress-signal to PP2A-Cdc55 appears to be transmitted via the protein kinase Rim15 that upon stress phosphorylates and activates the two PP2A-Cdc55 inhibitors Igo1 and Igo2 (Mochida *et al*. 2010). Overall, the authors demonstrate that one third of the stress-induced phosphorylome is under control of PP2A-Cdc55, making this phosphatase as impactful as Hog1. Thus, a signaling mechanism where the primary effect is the inhibition of a phosphatase instead of the activation of a kinase is the central effector component of this stress-induced signal transduction pathway (Hollenstein *et al*. 2021).

## THE CELL CYCLE AND OSMOSTRESS

Upon hyperosmotic challenge, proliferating cells delay cell cycle progression to allow time for cellular adjustments to prevent damage accumulation when progressing into sensitive phases of the cell cycle. Hog1 is one of the main players in this delay and upon activation, Hog1 rapidly and transiently migrates into the nucleus where it phosphorylates substrates that regulate cell cycle progression. The detrimental effects if these mechanisms are not operational are clearly seen in *hog1∆* cells that continue the cell cycle during osmostress, which results in cell death (Clotet *et al*. 2006; Jimenez *et al*. 2020).

The G1-to-S transition is regulated by Hog1 during hyperosmotic stress where sustained activation of the HOG pathway causes a prolonged arrest in G1 (Alexander *et al*. 2001; Escote *et al*. 2004). Mechanistically this arrest in G1 mainly works via stabilization of the cyclin-dependent kinase (CDK) inhibitor Sic1 and the downregulation of expression of the G1 cyclins Cln2 and Clb5. Hog1 phosphorylates Sic1 at threonine 173, which blocks its interaction with the E3 ligase Cdc4 leading to Sic1 stabilization. The importance of this Sic1-dependent arrest in the G1 phase for the osmoresponse is clearly seen in that cells lacking Sic1 or containing a Sic1 allele mutated in the Hog1 phosphorylation site thr173, are unable to arrest at G1 phase after osmotic stress and become osmo-sensitive (Escote *et al*. 2004). Hog1 also interacts with and phosphorylates components of the core cell cycle transcriptional machinery such as Whi5 and the coregulator Msa1 (Gonzalez-Novo *et al*. 2015). The Hog1-dependent phosphorylation of these two transcriptional regulators leads to inhibition of expression of the G1 cyclins, which appears essential for proper coordination of budding and DNA replication. In addition, Hog1 binds to the promoters of the G1 cyclins and in this direct way regulate their expression. Thus, Hog1 plays several important mechanistic roles in the cell cycle control of START.

During the S phase of the cell cycle osmotic stress adjustments impose challenges resulting from the enhanced transcription of a large set of genes (see below). During S phase transcription and DNA replication coexists in time and space and therefore must be coordinated to prevent transcription–replication conflicts. The collision between the machineries of replication and stress-induced transcription will result in replication fork stalling that leads to transcription-associated recombination i.e. genomic instability. In line with this it was shown that the increase in genomic instability during osmostress was caused by enhanced gene expression, since deletions of the main transcription factors responsible for the ESR (see below), Msn2 and Msn4, completely abolished the effect (Duch *et al*. 2018). It was also shown that Hog1 directly prevents collisions between the transcription and replication machineries by blocking DNA replication via phosphorylating the N-terminal region of Mrc1. Mrc1 is a regulatory component that links the helicase with DNA polymerase activities of the replicating complex. Several stresses, not only osmotic stress, provoked a delay in S phase that was mediated by the phosphorylation of Mrc1 at sites Thr169, Ser215, and Ser229 via several signaling kinases, Hog1 being responsible for the osmo-induced effects. Thus, Mrc1 integrates multiple external stimuli and constitutes a novel cell cycle checkpoint during S phase, called ‘Mrc1 transcription–replication safeguard mechanism’ (MTR), in relation to external adversity like hyperosmotic conditions.

Early reports suggested that osmostress also induces a delay in the G2 phase (Alexander *et al*. 2001). The G2-to-M transition is driven by the activity of the CDK-Clb2 complex, which is negatively regulated by Swe1 to ensure that cells have the required size to go through cell division. Cells remain in G2 until Swe1 is degraded, which is triggered by its phosphorylation by CDK-Clb2 or protein kinases Hsl1 and Cdc5. The activated Hog1 stabilizes Swe1 via phosphorylating the Hsl1 kinase at Thr169 and down-regulates the cyclin *CLB2*, which results in a transient arrest in G2 phase (Clotet *et al*. 2006), thereby allowing time for adaptation to osmostress.

Mitosis is another cell cycle phase in which a Hog1-dependent cell cycle control in response to osmostress exists. It was recently shown that activated Hog1 phosphorylates the core subunit of the RENT complex, Net1, at Thr62 and Ser385, and in that way alters its affinity for the dual-specificity phosphatase Cdc14 (Jimenez *et al*. 2020). The activity of Cdc14 is tightly regulated by its subcellular localization, and during most of the cell cycle Cdc14 is kept sequestered at the nucleolus by Net1. Phosphorylation of Cdc14 by CDK leads to release from Net1, upon which Cdc14 leaves the nucleolus. Different localization of the Cdc14 phosphatase allows its targeting of distinct substrates during anaphase progression. The Hog1-dependent phosphorylation of Net1 makes the Net1-Cdc14 complex more resistant to modifications by CDK and leads to that Cdc14 is sequestered at the nucleolus and mitosis is delayed (Jimenez *et al*. 2020). Consequently, the phosphorylation-negative mutant of Net1 (Thr62Ala, Ser385Ala), that cannot be phosphorylated by Hog1, displays reduced viability upon osmostress.

In summary, Hog1 contributes to maximizing cell survival upon stress by stopping the cell cycle in all cell cycle phases. This is interesting since other cell cycle control mechanisms act on one specific phase of the cell cycle. No wonder Hog1 deletion leads to severe effects in the cell cycle in response to osmotic stress.

## GENE EXPRESSION

### A large portion of the gene regulatory changes in response to osmostress is a result of a Hog1-mediated delay of the cell cycle

*Saccharomyces cerevisiae* rapidly and strongly regulates hundreds of genes' expression during an osmotic shock (Gasch *et al*. 2000; Causton *et al*. 2001). Many of these responses are similar irrespective of the type of stress applied, and this consistent transcriptional change has been termed the environmental stress response (ESR). Two large clusters of genes, one consisting of repressed genes (≈ 600) and one consisting of induced genes (≈300), display reciprocal but temporal profiles almost independent of the type of stress, jointly constituting roughly 14% of all genes. In fact, the expression response to 1 M sorbitol osmotic shock included only a few genes whose expression is specifically affected by this condition (Gasch *et al*. 2000), and among these the earliest and strongest responses were the induction of genes involved in the synthesis and regulation of glycerol and trehalose. A remarkable feature of the expression programs is that the change in transcript abundance is largely transient returning back to pre-shift levels after 60–120 minutes. In essence, the same response, with large number of genes and a transient response, is also seen during saline stress with NaCl (Posas *et al*. 2000; Causton *et al*. 2001) or KCl (O'Rourke and Herskowitz 2004). It was also reported that a large set of these genes were Hog1-dependent in their response to these two types of saline stress, but it is also clear that many genes showed a partial or fully Hog1-independent change in gene expression. This differential importance of Hog1 is in line with an earlier report based in two-dimensional analysis of ^35^S-labeled proteins after 20–40 min of exposure of the cells to 0.7 M NaCl, that many, but not all, of the observed changes in protein expression was dependent on the *PBS2* gene and thus the activation of Hog1 (Akhtar, Blomberg and Adler 1997).

In an elegant and ambitious time-resolved study of osmo-acclimation of various HOG pathway mutants, O'rourke and Herskowitz reported that > 500 genes (≈ 25% of all regulated genes) are dependent on Hog1 for osmotic regulation when exposed to 1 M sorbitol or 0.5 M KCl (O'Rourke and Herskowitz 2004). Clustering of these genes indicated six distinct gene expression patterns. However, only one of these clusters, containing 32 genes (the cluster contains ≈ 5% of the hog1-dependent genes), exhibits very strong induction in wild-type cells but no induction in the *hog1∆* strain. Some of the representatives in this class of fully Hog1-dependent genes are the sodium transporter *ENA1* and glycerol transporter *STL1*, the transketolase *TKL2*, the dihydroxyacetone kinase *DAK1*, and an enzyme involved in vitamin B1 biosynthesis, *THI4*. Two clusters contain genes induced in both wild-type and *hog1∆* mutant in response to high osmolarity, and among these HOG1-independent genes many of the general stress-response genes such as *CTT1*, *GRE1, HSP26, HSP12*, and *DDR48* are found, genes that have been shown to be strongly regulated by the transcription factors Msn2/4 (Rep *et al*. 2000). Alternatively, the constantly active Hog1 mutant HOG1^D170A+F318 L^ have been used to identify *bona fide* activated downstream targets of Hog1 even without applied stress (Bai, Tesker and Engelberg 2015). It was found that inducible expression of this intrinsically active Hog1 in *hog1Δpbs2Δ* mutant cells increased expression of roughly 100 genes, of which only about 1/10th was induced by at least 10-fold. Thus, there are a rather small number of genes for which Hog1 is sufficient for modifying their expression. In summary, this clearly supports that the osmo-responsive genes involve multiple regulatory components, in addition to Hog1.

In a study of the relation between growth rate and gene expression using steady-state continuous cultures limited by one of six different nutrients, it was found that the patterns of gene expression in relation to growth rate is strikingly similar for the six different media (Brauer *et al*. 2008). Thus, the expression of many genes are growth rate-dependent; a large group of genes increase their expression with increasing growth rate, and a comparably large group decrease their expression with increasing growth rate. The striking observation was that genes whose expression correlates with growth rate are highly represented among the genes generally responsive to stress (i.e. ESR genes). There is an overrepresentation of genes involved in translation (e.g. ribosomal components) that display higher expression at higher growth rates. This make sense since to grow at a faster rate, more proteins must be made per unit time effectuated by having more ribosomes per cell. In contrast, genes that are negatively correlated with growth rate were the ones being generally stress-induced, osmostress included. This raises the possibility that many of the ESR genes are not responding directly to a specific stress, but instead are responding to a reduction in growth rate secondary to the stress. In a follow-up study, it was found that the slow growth signature on gene expression was highly correlated with many published microarray datasets (O'Duibhir *et al*. 2014). In this context, the authors noted that many stressful conditions cause a transient G1 arrest. They therefore analyzed if a shift in the distribution of cells over different cell cycle phases can explain the ESR and found that cell cycle gene expression data closely resemble the ESR data. Thus, it was proposed that the ESR signature to a large extent reflects a cell cycle population shift, and in line with this they often found that the time resolved ESR data displayed a transient reduction in the pre-S phase marker *CLN2* cyclin. This also explains that the gene expression changes in response to hyperosmotic stress are transient—cells first end up in G1 where they adjust to the new stress-full conditions and then after acclimation start growing again by passing START and entering the S-phase. It also explains how Hog1 can have such drastic impact on the gene expression changes, given the deep involvement of Hog1 in control of the cell cycle progression during osmostress (see above). A general recommendation is given that this should be considered in any study of stress-perturbations that result in growth rate changes; subtracting the growth rate-dependent genes from the analysis will enhance the functional enrichment of the more stress-specific gene-responders.

### The transcriptional machinery and osmostress

The detailed mechanistic understanding of the control of osmo-regulated genes are in most cases still not fully deciphered, despite the fact that the entire regulatory region of most genes in *S. cerevisiae*, i.e. promoter and upstream activating sequences, is localized to a relatively short region only a couple of hundred base-pairs upstream of the start site of transcription (Struhl *et al*. 1998). Irrespective of the cause of these rapid inductions of gene expression during osmostress, they happen despite an in parallel overall reduction in transcriptional capacity with a general defect in transcription initiation in the first few minutes of stress (Proft and Struhl 2004). Thus, cells have evolved mechanisms to prioritize transcription of the most vital osmostress genes. How does this differential reallocation of the transcriptional machinery work? Much has been learnt about these mechanisms during the years and it is clear that the transcriptional activation of many osmostress genes is coordinated by Hog1 (Capaldi *et al*. 2008). Hog1 is rapidly localized to the nucleus in response to an osmotic stress where it regulates the action of several transcription factors and chromatin-modifying enzymes leading to re-allocation of the RNA pol II machinery to genes under osmotic control.

**Figure 4.**
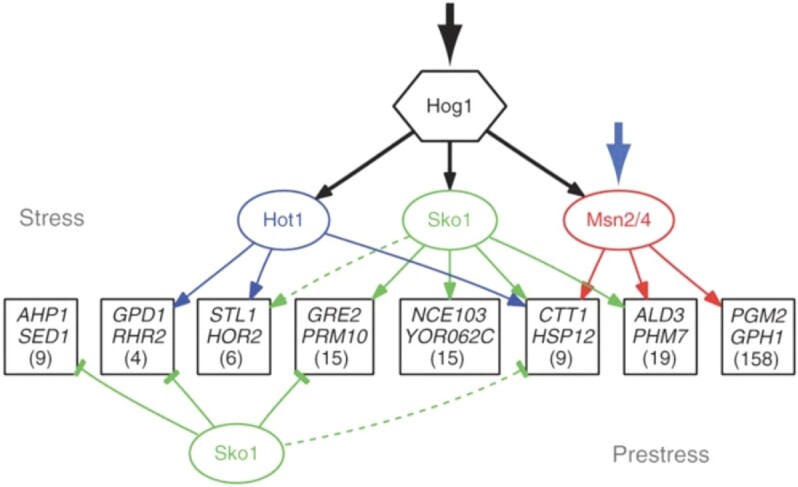
Model over the structure of the transcriptional network activated by Hog1 during KCl-induced osmotic stress. The model depicts that the incoming osmo-signal to Hog1 (thick black arrow) is spread out via Hog1 (medium-sized black arrows) to multiple transcription factors, Sko1, Hot1 and Msn2/4, that cooperated in different ways at different promoters (thin arrows in different colours). The general stress signal to the general transcription factor Msn2/4 (thick blue arrow) and the Hog-dependent signaling via the transcription factors Sko1 and Hot1 are integrated at a subset of the general stress–responsive genes. Targeted genes are grouped denoted by a box, where some genes are indicated by name and the numbers in parenthesis indicate the total number of genes in that group, on the basis of expression and common regulatory mechanisms. Groups are shown only if two or more genes have the same connections between the indicated transcription factors as determined by expression and confirmed by ChIP. Broken lines indicate interactions that exist for only some genes of a group. ‘Prestress’ indicates the regulatory mechanisms during no osmostress conditions (Sko1 repression), while ‘stress’ indicates the transcriptional mechanisms in operation during osmostress. This figure is from (Capaldi *et al*. 2008) and is published with permission from Springer Nature.

Applying large-scale gene expression and DNA binding assays together with epistatic analysis analyzing single and double mutants, suggested that most Hog1-dependent genes are regulated in single or in combinations with the transcriptional factors Msn2/4, Hot1 and Sko1 (Capaldi *et al*. 2008). A model was proposed where activated Hog1 impacts on multiple transcription factors and then cooperate in different ways at different promoters (Fig. 4). Initially, only analyzing Hog1 and Msn2/4, they found that these two factors cooperate at most genes in the network, however, these two factors can also work independently. It was proposed that Hog1 might activate Msn2/4 through phosphorylation at one or more of ≈ 10 MAPK consensus sites found in Msn2/4, or indirectly through the other kinases, phosphatases and 14–3-3 proteins that regulate Msn2/4 nuclear import and export (Garreau *et al*. 2000). Incorporation of also Sko1 and Hot1 into the network model it was revealed that induction by Hog1 is almost entirely through Sko1, Hot1 and Msn2/4 since these factors are required for 88% of Hog1-dependent gene activation. Chromatin immunoprecipitation for these transcription factors and subsequent identification of target promoters, showed that > 65% of genes were bound by the appropriate factor. The factor-promoter connection was further supported by the presence of regulatory motifs in the target-genes’ promoters. By comparing the response during different types of stress, high glucose or high salt (at the same osmolarity), they found that the transcriptional program activated by Hog1 is context dependent. In glucose-imposed osmostress roughly 70 genes controlled by Hog1 alone were activated, however, in this condition cells did not induce the 200 general stress genes that are Msn2/4-dependent. Instead, in high glucose a subset of the Msn2/4-dependent genes where Sko1/Hot1 and Msn2/4 cooperate was induced, further supporting their mechanistic model for the regulation of Hog1-dependent genes (Fig. 4). Hog1 phosphorylates Hot1, however, this phosphorylation is not essential for Hot1 transcriptional activity (Alepuz *et al*. 2003). It is instead hypothesised that Hot1 associates physically with its target promoters where it during osmostress binds active Hog1. In this way it recruits Hog1 to the promoter where Hog1 functions as a transcription factor by recruiting the chromatin-remodeling component Rpd3, the RNA Pol II and the mediator complex (Alepuz *et al*. 2003; De Nadal *et al*. 2004).

**Figure 5.**
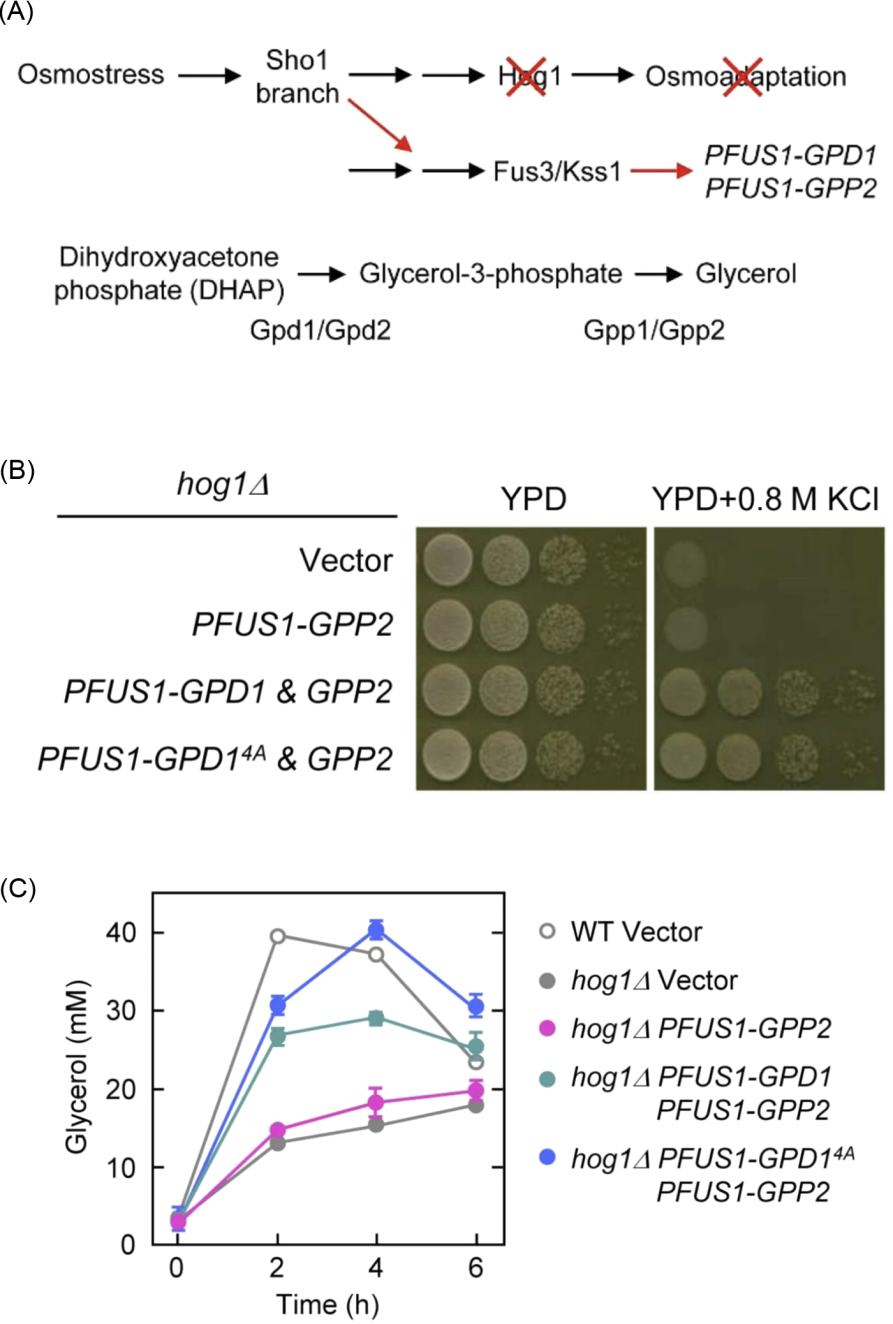
‘Synthetic’ osmoadaptation in *hog1∆* cells where expression of Hog1-dependent osmostress-induced genes (*GPD1* and *GPP2*) was rewired under the control of a Fus3/Kss1-dependent *FUS1* promoter. **(A)** Experimental design for synthetic osmoadaptation in *hog1∆* using crosstalk for the production of the key-enzymes Gpd1 and Gpp2 in the glycerol biosynthesis pathway. **(B)** Synthetic induction of *GPD1* and *GPP2* strongly suppresses osmosensitivity in YPD + 0.8 M NaCl medium of the *hog1∆* strain. GPD1^4A^ stands for the unphosphorylated form of Gpd1 (four serines in the N-terminus converted into alanine) with a constitutively high enzymatic activity. **(C)** The intracellular accumulation of glycerol in the strains in B. This figure is from (Babazadeh *et al*. 2014).

RNA Pol II and stress-specific transcription factors must overcome the presence of nucleosomes for efficient transcription. During osmostress in yeast dramatic changes occur in the nucleosome organization of stress-responsive promoters, which is dependent on Hog1 and the RSC chromatin structure-remodeling complex (Mas *et al*. 2009). It was shown that Hog1 physically interacts with RSC to direct its association with osmo-responsive genes. In addition, the importance of this was seen in RSC mutants resulting in reduced stress gene expression and higher osmo-sensitivity. In this context it is also interesting to note that the *GPD1* gene is regulated by the activator/repressor protein Rap1 during exponential growth under hyperosmotic conditions (Eriksson *et al*. 2000). By promoter deletions it was shown that a region positioned between -478 to -324 nucleotides upstream start of translation is important for both basal activity and osmotic induction of *GPD1*. This region contains three consensus sequences for Rap1p-binding that also was shown functional *in vivo*. However, the detected Rap1p-DNA interactions were not affected by changes in the osmolarity of the growth medium. In contrast, inactivation of the individual Rap1p-binding sites by point mutations in the consensus binding sites strongly hampered osmotic induction. Interestingly, the different binding sites were differentially important for low- and high- salinity. Rap1 can bind to various protein-coding genes to regulate their expression (Lieb *et al*. 2001). ChIP-seq analysis has revealed that the transcription factor Fpr1 binds to the promoters of ribosomal protein genes in a Rap1-dependent manner (Kasahara *et al*. 2020). Similarly, one can envisage that *GPD1* transcription factors like Hot1 and Hog1 might bind to the promoter in a Rap1-dependent fashion. It was recently shown that Rap1 can invade compact chromatin fibers and directly open chromatin structure for protein coding genes (Mivelaz *et al*. 2020). Rap1 is thus acting as a ‘pioneer transcription factor’ which together with RSC displace nucleosomes to generate an active promoter state.

After a hyperosmotic stress the Rpd3 histone deacetylase complex is recruited to stress-activated promoters by binding to Hog1. Hog1 targets the Rpd3–Sin3 complex to specific promoters which results in histone deacetylation, entry of RNA polymerase II and induction of gene expression. It was found that cells lacking the Rpd3–Sin3 histone deacetylase complex show compromised expression of osmostress genes and are sensitive to high osmolarity. Besides acetylation/deacetylation, other histone post-translational modifications are associated with transcriptional activation or repression. By high-throughput screening, transcription initiation in response to stress has been measured at the single cell level for a library of > 500 histone point-mutants that covers the fours histones H3, H4, H2A and H2B (Vieitez *et al*. 2020). The transcriptional output was measured for two different strong osmostress promoters, *pALD3* and *pSTL1*. It was found that many histone-mutants induced the same transcriptional defects on both *pALD3* and *pSTL1*. A substitution in one of these mutations on histone H4, H4-S47D (the mutation of the serine 47 to an aspartic acid) resulted in a reduced expression of *pALD3* upon osmostress, whereas the non-phosphorylatable alanine substitution (H4-S47A) had no effect on the expression of this reporter. It was found that the protein kinases Cla4 and Ste20 were involved in this H4-S47 phosphorylation, and both protein kinases were also shown to be recruited to the *ALD3* promoter during osmostress. Even if this data might not explain the regulation of osmo-responsive genes at this stage, it gives an interesting glimpse of the additional complexity, besides the network of transcription factors, that underlays osmostress gene regulation.

The *STL1* gene was picked up as one of few genes being strongly regulated in a strain expressing the spontaneously active variant Hog1^D170A+F318 L^ in cells lacking the Hog1 activator Pbs2 (Bai, Tesker and Engelberg 2015). By promoter swapping/truncation studies the authors identified a novel osmostress- and Hog1-regulated cis-element called the Hog1-responsive element [HoRE] that contains two short identical repeats of the sequence 5′-CATTTGGC-3′ and a similar third repeat. They furthermore show that this element binds Hot1 *in vitro*. The interdependence of Hog1 and Hot1 in the regulation of *STL1* was shown by the fact that overexpression of Hog1^D170A+F318 L^ in *hot1Δ* cells under hyperosmotic conditions did not activate the *STL1* promoter, and similarly, overexpression of Hot1 in *hog1∆* cells was unable to activate *STL1*. In addition, they found a putative Sko1-binding site in the full-length HoRE element, however, *pSTL1* activity was totally abolished only in *hog1Δ* and *hot1Δ* while being reduced 5-fold in *sko1Δ*. Surprisingly, they did not find the HoRE element in any of the other strong osmostress genes, and the only gene whose expression was abolished in *hot1Δ* cells was *STL1*. Thus, the authors propose that Hot1 may be a transcription factors that are essential for transcription of very few genes or even just one. A recent follow-up study from the same research group, in this case focusing on mechanisms behind on Hog1-dependent genes that are not so strongly induced under osmostress (*RTC3*, *HSP12*, *DAK1* and *ALD3*), reported that all four promoters were regulated to different degrees by Msn2/4, including *DAK1*, which does not contain any stress-responsive elements (STREs) in its promoter (Bai *et al*. 2020). They found that *DAK1* and *ALD3* promoters were dependent on a single activator, *DAK1* on Sko1 and *ALD3* on Msn2/4. In addition, two other genes, *RTC3* and *HSP12*, were induced even in *msn2Δmsn4Δsko1Δhot1Δ* cells lacking all the known osmo-regulators, indicating that some novel mechanism must be involved. In addition, it was also reported that *msn2Δmsn4Δhot1Δsko1Δ* cells, lacking all four main transcription factors that are currently known to be involved in the activation of osmo-regulated genes, are not sensitive to osmostress.

The bZIP transcription factor Sko1 was first characterized as a repressor of genes which promoter contains the cAMP response element (CRE), e.g. the osmo-induced *ENA1* (Proft and Serrano 1999). The repression by Sko1 involves recruitment of the Tup1/Cyc8 corepressor complex to these promoters. During osmotic stress the repression is relieved in response to phosphorylation of Sko1 by Hog1 (Proft *et al*. 2001). Besides de-repression, Sko1 has also been reported to play a role as an activator in the induction of some stress-responsive genes, through the Hog1-dependent recruitment of the SAGA and SWI/SNF nucleosome remodelers to promoters bound by the Sko1/Tup1/Cyc8 complex (Proft and Struhl 2002). An additional factor that appears to play a role in the specificity of Sko1 for stress-responsive promoters was recently shown to be via SUMOylation on residue Lys 567 (Sri Theivakadadcham, Bergey and Rosonina 2019). This modification leads to the covalent attachment of a ∼12-kDa SUMO (Small Ubiquitin-like Modifier) peptide to specific lysine residues on proteins through a process analogous to ubiquitination. Sequence-specific transcription factors represent one of the largest groups of proteins targeted for SUMO post-translational modification and it has been demonstrated to be present at induced genes (Chymkowitch *et al*. 2015). Even if Sko1 are often targeted to osmoresponse genes, it was found that the Sko1 sumoylation is not stress-regulated. Furthermore, the modification does not dependent on phosphorylation by Hog1. Sko1 mutants that cannot bind DNA are devoid of sumoylation but restoring DNA binding by a heterologous DNA binding domain restores the modification; DNA binding is thus a major determinant for Sko1 sumoylation (Sri Theivakadadcham, Bergey and Rosonina 2019). A sumoylation-deficient Sko1 mutant displays increased occupancy at many of its binding sites, which was found to inhibit the Hog1 recruitment to some induced osmostress genes. A working hypothesis was proposed for a general role of sumoylation in reducing the association of transcription factors with chromatin. More research will be needed to fully understand the role of this interesting modification in gene regulation during hyperosmotic conditions.

In addition, transcript stability also plays a role in setting the level of osmo-instigated change in gene expression. The dynamic transcriptional response was analyzed via uptake and metabolic RNA labeling by the nucleoside analog 4sU, allowing highly time-resolved measurements of RNA synthesis rate and RNA half-lives during acclimation to hyperosmotic conditions (Miller *et al*. 2011). Three distinct phases of the stress response were reported; (i) in the initial shock phase mRNA synthesis and decay rates decrease globally, (ii) in the subsequent induction phase both rates increase for a subset of genes, and (iii) in the recovery phase mRNA decay rates are largely restored, whereas synthesis rates remain altered. Thus, there is an interplay between changes in synthesis rate and decay rate of mRNA during osmotic stress. An interesting new paradigm proposes crosstalk between nuclear transcription and cytoplasmic mRNA stability in setting the expression levels of genes. In a recent study of the role of the 5'–3' mRNA exonuclease Xrn1 in the regulation of transcription, genomic run-on (GRO) analysis was performed to examine Xrn1’s time-resolved effect on transcription during osmotic adjustment (Garcia-Martinez *et al*. 2021). It was shown that Xrn1 is necessary to maintain proper kinetics of regulated genes, without any differential effect on genes being dependent on the transcription factors Hot1, Sko1, or Smp1 (that all act down-stream of Hog1) for their osmotic induction. For selected osmotic stress-upregulated genes i.e. *STL1* and *GRE3*, transcription was strongly reduced in the *xrn1Δ* mutant, however, their corresponding mRNAs were stabilized. The authors conclude that Xrn1, besides being an exonuclease with an impact on transcript stability, acts as a transcription elongation factor by binding to upregulated genes in a Hog1-dependent manner preventing the accumulation of inactive ‘backtracked’ RNA polylmerase II. However, Xrn1 appears to play a more general role in transcription and not being selectively involved in regulating osmotic-stress genes.

### The regulation of osmo-genes appears carbon source dependent

It has been shown that the Hog1 kinase is activated during osmotic stress in the same way irrespective of carbon and energy source; Hog1 phosphorylation and nuclear residence in response to increased salinity are very similar for growth on glucose and ethanol and the *hog1Δ* mutant is also osmo-sensitive during growth on either carbon source (Babazadeh *et al*. 2017). This study also tested the effect of some key stress-regulators on the expression of genes that are strongly upregulated during osmostress, i.e. *STL1, ALD2, GPD1, HSP12*, and *TPS1*, and all of these were strongly affected by deletion of *HOG1* on both carbon sources. However, it was noted that all genes showed osmostress-mediated upregulation in *hog1∆* mutant that was strongest in ethanol medium, thus, indicating that Hog1-independent mechanisms are activated during respiratoric metabolism. The authors also tested two other main regulatory systems during stress, Msn2/4 and PKA, and found that neither abolished the osmotic induction of the genes. However, these two regulatory systems were found to influence the level of expression of a subset of the tested genes, while the salt-inducted levels of *STL1* and *GPD1* were totally invariant to manipulations of these factors. Control of gene expression of osmo-genes acts via an interplay between different pathways and components, a situation that appears to be even more complex for ethanol-grown cells.

## POST-TRANSLATIONAL MODIFICATIONS AND THEIR IMPACT ON GLYCEROL PRODUCTION AND ACCUMULATION

### Glycerol production

Cytoplasmic glycerol-3-phosphate dehydrogenases (GPDs) catalyze the NADH-dependent reduction of dihydroxyacetone phosphate (DHAP) to glycerol-3-phosphate. This reaction is the first step in the biosynthetic pathway diverting from glycolysis and leading to glycerol production (Hohmann 2015). The enzyme GPD is important in a number of contexts for *S. cerevisiae*: (i) during exponential growth GPD maintains cytoplasmic redox balance by reoxidizing NADH produced in the lower part of glycolysis by glyceraldehyde 3-phosphate dehydrogenase, (ii) under hyperosmotic stress glycerol accumulates intracellularly and plays the role of an essential osmolyte, (iii) GPD participate in a mitochondrial NADH shuttle, iv) GPD reduces the production of the toxic metabolic product methylglyoxal through disposal of its precursor DHAP, and (iv) glycerol-3-phosphate is a precursor in phospholipid biosynthesis.

GPD is encoded by two paralogous genes, Gpd1 and Gpd2, that have only partially overlapping *in vivo* functions. Acclimation to hyperosmotic conditions with enhanced glycerol production is more dependent on *Gpd1*, while mutants lacking *GPD2* show poor growth during anaerobic conditions (Ansell *et al*. 1997). *GPD1* and *GPD2* are differentially regulated at the transcriptional level, with *GPD1* being the main form upregulated in response to hyperosmotic stress and *GPD2* expression being stimulated by hypoxia. *GPD1* is also regarded to be the key transcriptional target of the Hog1 kinase. The distinct functions of the two GPDs has been attributed in part to differential protein compartmentalization: Gpd2 localizes to the cytosol and mitochondria, while Gpd1 is found in the cytosol and in peroxisomes.

It would seem to make good physiological sense that the initial defense to acute hyperosmotic stress should not depend on slow processes, like transcription and translation, but rather should act primarily through immediate effects on preexisting proteins. MS-based phosphoproteomics manifested that Ser24 and Ser27 in the Gpd1 N-terminus are phosphorylated, and that the enzyme can occur in both single and double phosphorylated forms (Oliveira *et al*. 2012). The importance of phosphorylation at these sites and the two nearby sites Ser23 and Ser25 was proven since serine to alanine mutants lacking all these four phosphorylation sites exhibited two-fold higher glycerol yield and two-fold higher glycerol-3-phosphate dehydrogenase activity. Thus, phosphorylation in its N-terminal part inhibits Gpd1 activity. However, it appears that besides Ser24 and Ser27 the neighboring serines Ser23 and Ser25 can also be phosphorylated and play a role in the inhibition of Gpd1 activity, since the double-point mutant Gpd1[S24A/S27A] did not display altered glycerol yield or enzyme activity.

The activity of both the Gpd1 and Gpd2 enzymes are negatively regulated through phosphorylation at a conserved site in their respective N-termini by distinct kinases, thereby enabling rapid acclimation to specific stress conditions. While Gpd2 is phosphorylated and partially inactivated by the AMP-activated protein kinase Snf1 under glucose-limiting conditions, which leads to less glycerol produced and the potential fine-tuning of the carbon flow, Gpd1 is phosphorylated by the TORC2-dependent kinases Ypk1 and Ypk2 (Lee *et al*. 2012). The serine/threonine kinase Ypk1 gets inactivated under osmostress and the resulting dephosphorylation of Gpd1 then leads to increased GDP activity and thus enhanced glycerol production. It was also demonstrated that TORC2-dependent phosphorylation of Ypk1 at Thr662, a modification required for maximal activation of Ypk1, is inhibited during hyperosmotic shock. Importantly, also in a *hog1∆* strain both the inactivation of Ypk1 and the Gpd1 dephosphorylation occur, showing that downregulation of TORC2-dependent phosphorylation of Ypk1 does not require Hog1.

Finally, it should be noted that Gpd1 becomes dephosphorylated by a currently unknown protein phosphatase during osmostress conditions. However, in a recent phosphoproteomics study where a large set of kinase and phosphatase deletion mutants were investigated proteome-wide for changes in phosphorylation on potential target proteins, they found a roughly 4-fold increase in the phosphorylation of serine 24 of Gpd1 in the *sit4∆* strain (Li *et al*. 2019). This suggests that the type 2A-related serine-threonine phosphatase Sit4 is a potential candidate for such a phosphatase that might directly influence the phosphorylation status of Gpd1 together with the Ypk1 kinase. In this context it is interesting to note that another Ypk1 substrate, the protein kinase Fpk1 that is negatively regulated via phosphorylation by Ypk1, is dephosphorylated and reactivated by Sit4 in complex with its co-activator Sap190 (Roelants *et al*. 2015).

### Glycerol accumulation

To regulate intracellular glycerol, yeast employs the glycerol channel Fps1 (Luyten *et al*. 1995; Ahmadpour *et al*. 2014). The Fps1 channel appears to be the main efflux system for glycerol since deletion of *FPS1* renders yeast cells sensitive to hypoosmotic shock and to anaerobicity, and it has been shown that intracellular glycerol is elevated in *fps1Δ* cells even in the absence of hyperosmotic stress (Tamas *et al*. 1999). Fps1 is an unusual aquaglyceroporin with long N- and C-terminal extensions that are required for Fps1 closure (Ahmadpour *et al*. 2014). It has been reported that two sites in the N-terminal part of Fps1 (Ser181 and Thr185) undergo changes in phosphorylation during hyperosmotic stress (Kanshin *et al*. 2015).

Hog1 negatively affects the function of the aquaglyceroporin Fps1 via phosphorylation of the co-activator Rgc2 (and probably also Rgc1) that leads to its displacement from Fps1, which then closes the channel and prevents glycerol efflux (Lee *et al*. 2013). In a recent phosphoproteomics study during hyperosmotic stress it was documented that the phosphorylation of Thr774 and Ser975 of Rgc1 increased roughly 10-fold during osmostress, while Thr808 of Rgc2 increased roughly 3-fold (Romanov *et al*. 2017). In this study it was also confirmed that there is a decrease in phosphorylation of Ser185 on Fps1, a site that has been proposed to activate docking of Hog1 to this channel (Lee *et al*. 2013). Fps1 can also be negatively regulated by Hog1 via phosphorylation that stimulates its internalization and degradation (Thorsen *et al*. 2006). However, Hog1 seems to be dispensable for Fps1 closure after hyperosmotic shock (Luyten *et al*. 1995) indicating that there would exist alterative mechanisms for channel closure.

One alternative signaling pathway that controls Fps1 has been unraveled by Jeremy Thorner and his group who have shown that Fps1 is also a substrate of the serine/threonine kinase Ypk1. The Ypk1-dependent phosphorylation of Fps1 was initially identified in a genome-wide screen for TORC2-Ypk1 substrates (Muir *et al*. 2014). The modification's functional importance was later verified to lead to the open channel state of Fps1 and that the Ypk1-dependent closure of Fps1 during hyperosmotic stress is Hog1-independent (Muir *et al*. 2015). The functional importance of phosphorylations in the N-terminus of Fps1 was verified by serine-to-alanine substitutions at all three sites (Fps1-3A) inhibiting phosphorylation at these sites and showing that the Fps1-3A *hog1Δ* strain accumulated more glycerol and was also more osmo-resistant than *hog1Δ*. Thus, closure of the Fps1 channel by lack of Ypk1 phosphorylation occurs independently of any effects requiring Hog1. Furthermore, they found that loss of TORC2-mediated Ypk1 phosphorylation occurs very rapidly (within 1 min) of hyperosmotic shock. However, the lack of Fps1 phosphorylation is transient and the TORC2-Ypk1-mediated phosphorylation was again detectable almost at pre-stress level after 75 min. This is surprising since intracellular glycerol accumulation persists during the whole growth cycle, and the resumed Fps1 phosphorylation would indicating that Fps1 is in its open state throughout growth under osmostress. Thus, the TORC2-Ypk1-dependent initial rapid closure of Fps1 appears to later be taken over by some other long-term mechanism. In conclusion, the presence of different systems—TORC2-Ypk1 and Hog1, as well as potentially other systems—that regulates proteins involved in glycerol production and accumulation might allow cells to adjust optimally and rapidly to different types, intensities and durations of osmostress.

### Hog1 and glycerol—the key molecule

The fact that Hog1 enters the nucleus to induce changes in gene expression of > 50 genes has been seen as essential for proper cellular adjustments and growth under hyperosmotic stress. However, this HOG-centric view on gene expression and osmoregulation has also been challenged. In an elegant study, Westfall et al. established that the Hog1-mediated transcriptional response is not essential for resistance to hyperosmotic stress (Westfall *et al*. 2008). This study was inspired by their surprising finding that mutants lacking Nmd5, the karyopherin previously shown by others to be necessary for Hog1 to enter the nucleus (Ferrigno *et al*. 1998), were resistant to hyperosmotic conditions. Hence, to further test whether nuclear entry of Hog1 is required for survival under hypertonic conditions, two types of constructs were made that both tethered Hog1 to the plasma membrane, either via appending C-terminal residues of Ras2 that are targets for attachment of S-palmitoyl and S-farnesyl (which anchors Hog1 in the membrane) or by fusion to residues 1–296 of the alpha-factor receptor Ste2 making the Hog1-fusion an integral membrane protein. The membrane-tethered Hog1 was plasma membrane-localized both during normal conditions and after hyperosmotic shock. As expected, the membrane-tethering of Hog1 abolished gene expression of known Hog1-dependent osmostress genes (*ALD3*, *CTT1*, *GPD1*, and *STL1*). Most strikingly, despite the lack of altered gene expression the membrane tethered Hog1 permitted good growth under hyperosmotic stress. In addition, deletion of transcriptional regulators reported to mediate Hog1-dependent gene regulation in response to hyperosmotic shock i.e. Hot1/Msn1, Sko1, Msn2/4 and Smp1 in the strain with the tethered Hog1, did not result in osmosensitivity. In summary, these results suggest that the well-documented ability of Hog1 to confer osmoresistance does not depend on transcriptional regulation. However, it was shown that the growth response was dependent on Hog1 catalytic activity indicating there are vital cytoplasmic target(s) that are phosphorylated. This study-design was repeated in a recent single-cell study with promoter-reporters for stress-responsive genes e.g. *STL1* and *GPD1* (Wosika and Pelet 2020). Applying the same logic as Westfall et al. with anchoring Hog1 to the plasma membrane by a fusion to the C-terminal residues of Ras2, they found that the activity of the promoter of *STL1* was abolished while the *GPD1* promoter was barely affected. For both these studies it cannot totally be rolled out that a small fraction of Hog1 might escape tethering and enter the nucleus, where some highly Hog1-dependent promoters might respond. However, while Westfall et al. looked at the expression from the intact *GPD1* and *STL1* genes, Wosika et al used reporters governed by only the promoters of these two genes. Thus, any kind of regulatory elements outside the *GPD1* promoter would have been missed in the latter study. It should also be noted that the two studies used different types of osmostress agents, sorbitol versus NaCl, which might influence the results. In any case, these Hog1-teathering studies are highly interesting and certainly deserves further future investigations.

Crosstalk exists between different signaling pathways, with the Fus3/Kss1 pathway being improperly activated in *hog1∆* mutants upon osmostress. This fact was utilized by Babazadeh et al in a study to induce *GPD1* and *GPP2* independently of Hog1, by placing these two glycerol-producing genes under Fus3 control via the promoter of *FUS1* (*pFUS1-GPD1*; *pFUS1-GPP2*) in *hog1∆* cells (Babazadeh *et al*. 2014). They found that up-regulation of only these two Hog1-dependent glycerol biosynthesis genes, *GPD1* and *GPP2*, was sufficient for successful suppression of osmo-sensitivity in *hog1∆* mutants (Fig. 5). Osmostress growth of these transformants correlated well with their ability to accumulate glycerol. This is in line with earlier studies which reported that overexpression of *GPD1* partly suppresses the hyper-osmosensitive phenotype of the *hog1∆* mutant (Albertyn *et al*. 1994). In summary, these results reveal that the role of Hog1 in the osmostress response under the studied conditions is not primarily its effect on transcription, but rather its role in an osmo-related delay in the cell cycle (see above) to allow time to establish elevated glycerol levels in combination with its role as a protein kinase with cytoplasmic targets that are vital for osmotic adjustments.

### Beyond glycerol—trehalose

It has been reported that the osmo-response in yeast can be strongly carbon source dependent (André, Hemming and Adler 1991; Babazadeh *et al*. 2017). Most strikingly, these studies show that *S. cerevisiae* respiring ethanol as the sole carbon and energy source does not substantially accumulate glycerol as the osmolyte under osmostress. Instead, under these fully respiratory conditions the intracellularly concentration of the disaccharide trehalose increases during osmotic stress. However, the rise in trehalose does not fully osmotically compensate for the lack of glycerol since the hyperosmotic increase in glycerol is ≈ 10 molar units while the increase in trehalose during growth on the corresponding salinities but utilizing ethanol is ≈ 2–4 molar units (the response is slightly strain dependent)(Babazadeh *et al*. 2017). It was concluded that some other osmolyte, presently not known, will have to complement trehalose to fully restore the osmotic potential in *S. cerevisae* during these hyperosmotic and respiratory conditions. However, it cannot be fully excluded that ethanol-grown cells would have changed requirements for osmolyte accumulation, implying that the osmotic balance/turgor pressure to be established doesn't have to be identical during hyperosmotic conditions when growing on different carbon sources. Indeed, changes in yeast cell wall properties like thickness and elasticity that could influence turgor have been documented in relation to the utilized carbon source [discussed in (Elhasi and Blomberg 2019)]. However, what talks against a carbon-source dependent change in osmotic behavior of cells is that glucose- and ethanol-grown cells behaved similarly and rapidly lost about 25%–30% of their volume upon osmotic shock (Babazadeh *et al*. 2017). Alternatively, one might speculate that the already high intracellular trehalose content during growth on ethanol in control/no osmostress medium, would constitute an osmotic buffer if the trehalose could go from a bound (not osmotically active) to a free (osmotically active) state in response to hyperosmotic stress.

Although the most stress-related role of trehalose is accumulation during heat stress where it functions as a small chemical chaperone (Verghese *et al*. 2012), another established mechanistic role of trehalose is linked to preservation of membrane structures (Crowe, Crowe and Chapman 1984) a mechanism that could also be relevant during osmotic dehydration. In addition, a number of phospho-sites in the regulatory subunit Tsl1 of the trehalose 6-phosphate synthase/phosphatase complex as well as the trehalase Nth1 that both take part in trehalose metabolism (production and break-down, respectively) have recently been shown to display altered levels of phosphorylations at specific sites during hyperosmotic stress (Romanov *et al*. 2017). Some of these sites were identified as direct targets of Hog1 thus indicating a connection between Hog1-signaling and trehalose metabolism. This is in line with that a mutant lacking *HOG1* is osmo-sensitive both on glucose and ethanol medium, and Hog1 phosphorylation and nuclear residence following treatment with NaCl were similar for growth on either carbon source, showing that the importance of Hog1 appears independent of major catabolic changes (Babazadeh *et al*. 2017).

## FUTURE CHALLENGES—LOOSE ENDS

### Many not so well understood mechanisms

This review has by no means covered all the interesting aspects of the yeast osmotic response but has outlined current views on the main features of the game—sensing mechanisms, signaling pathways (HOG-dependent and HOG-independent), transcriptional responses and glycerol production/accumulation. However, there are many important osmoregulatory aspects that are HOG-independent (Saxena and Sitaraman 2016) that have not been covered. Some of these functional aspects are ion transport over vacuolar membranes, Ca^2+^ handling and the calcineurin/calmodulin regulatory system, crosstalk between HOG and other signaling systems (only touched upon briefly in this review), and uptake and use of other osmolytes like amino acids, to mention a few. A clear sign of that the cellular osmo-response is nothing but complex is the fact that more than 200 gene knock-outs display a growth defect even in a rather moderate NaCl concentration (0.85 M NaCl)(Warringer *et al*. 2003). In this type of genome-wide phenotypic screen the expected ‘suspects’ are being revealed as osmo-sensitive, but in addition also many not so well studied protein-encoding genes like *YDR119w* (encodes a putative amino acid permease), *YJL184w/Gon7* (EKC/KEOPS protein complex required for tRNA modification), and *YLR097c/Hrt3* (putative SCF-ubiquitin ligase F-box protein). Looking at these lists of interesting but not so easy to explain osmo-genes clearly reveal that we still have some distance to go to reach the complete/genome-wide integrative description of the osmo-response. There are reasons to believe that glycerol and glycerol-related processes will stand the test in the long run and remain in the mechanistic center for osmotic adjustments, and some of the currently un-explained osmo-genes might indirectly connect to glycerol production/accumulation in some way. However, there will certainly be a rather wide array of interesting and fruitful functional ‘rockies’ to explore in the future in the light of osmotic stress.

### The osmotic response is conditional

Another thing that would be worth-while pursuing is the conditional nature of mechanisms in osmoregulation. It has been reported that osmotolerance in yeast is lower upon growth on the less fermentable carbon source galactose, and this was accompanied by a reduced accumulation of glycerol (Vanacloig-Pedros *et al*. 2015). This follow what was discussed above about the devoid of glycerol accumulation and instead enhanced levels of trehalose in relation to an altered carbon source—ethanol instead of glucose (Babazadeh *et al*. 2017). This behavior in *S. cerevisiae* resembles the temporal use of different osmolytes in the marine yeast *Debaryomyces hansenii* (Adler, Blomberg and Nilsson 1985). This marine yeast uses glycerol as the sole osmolyte during the earlier phases of growth, but then shifts to the production and accumulation of the polyol arabinitol when entering the stationary phase. The rational for this phenomenon is believed to be diminished leakage and thus it would be energetically favorable with accumulation of the five-carbon arabinitol instead of the three-carbon glycerol. If similar arguments would hold for the *S. cerevisiae* production and accumulation of trehalose instead of glycerol when grown on ethanol would be worth-while to study. Or does the explanation more relate to altered main metabolism during respiration that makes glycerol production a less efficient solution? In this context it would be interesting to study the connection between *GPD1* and *PNC1*, which is a nicotinamidase that converts nicotinamide to nicotinic acid and is part of the NAD^+^ salvage pathway. Pnc1 is thus linked to the production of one of the substrates (NADH) for the Gpd1 enzyme and glycerol production, and its overall gene expression profile is very similar to *GPD1* (see SGD website on expression similarities). How would these systems cooperate during diverse metabolic regimes, in particular during other reductive conditions (like anaerobiosis)? It would be interesting to see if *S. cerevisiae* would take up and use externally added minor amounts of glycerol during respiratory growth? Relevant in this regard is also the finding that anaerobic conditions, that promote glycerol production even under no osmotic stress, leads to faster and more efficient osmoregulation via glycerol under hyperosmotic conditions (Babazadeh *et al*. 2017).

### Short-term and long-term signaling and transcriptional responses

An overwhelming number of studies concerning transcriptional changes during osmotic stress concerns short-term responses—what happens over the first hour or so of acclimation/adaptation to osmotic stress. One of the reasons for this is certainly the strong gene expression responses with high levels of inductions, with in many cases an almost 100-fold increase in expression. However, it should be emphasized that almost all these responses are transient and after some time expression levels are back to, or almost back to, pre-shift levels. What does this mean? An overshoot in expression response with great increases in transcript levels will of course shorten the time it takes for cells to produce the needed new proteins and adjust physiology to start to grow again. Thus, after the initial adjustments when growth re-starts it is apparently sufficient with rather modest changes in expression to support growth and division for many, if not to say an infinite, number of doublings. However, the mechanisms involved during acclimation to osmostress, might not be identical to the ones that will dominate during proliferation and steady-state growth. It is clear from gene knock-out studies that there will be mechanistic differences, since some genes that are important in one phase of growth (the knock-out shows a phenotypic defect) will not be important for other growth phases (Warringer *et al*. 2003). I would therefore propose that a pressing aspect to investigate is what transcriptional and signaling mechanisms are important during long-term osmo-adjustments and exponential growth, now when we have a rather deep understanding of important features during acclimation in the lag-phase.

## Funding

Work in my laboratory on osmoregulation has over the years been supported by the Swedish Research Councils VR (2017-04559) and FORMAS (2013-549), as well as the European Commission (FP7 project UNICELLSYS; No. 201142 to Stefan Hohmann).

## Notes

In memory of Stefan Hohmann.
